# Mesalazine treatment causing resolution of intracranial hypertension secondary to ulcerative colitis: A case report

**DOI:** 10.1097/MD.0000000000013365

**Published:** 2018-12-10

**Authors:** Raoul Kanav Khanna, Rabih Hage, Alexandre Hage, Vanessa Polin, Thomas Sené, Catherine Vignal-Clermont

**Affiliations:** a Department of Ophthalmology, Fondation Ophtalmologique Adolphe de Rothschild; b Department of Gastroenterology, Hôpital de la Croix Saint-Simon; c Department of Internal Medicine, Fondation Ophtalmologique Adolphe de Rothschild, Paris, France.

**Keywords:** inflammatory bowel disease, intracranial hypertension, mesalazine, ulcerative colitis

## Abstract

**Rationale:**

The association between intracranial hypertension (ICH) and ulcerative colitis (UC) is rare. We report the unusual case of a male patient with UC and ICH in whom both conditions resolved with mesalazine therapy.

**Patient concerns:**

A 48-year-old Caucasian man presented to our department in June 2016 for decreased vision, transient visual obscuration, pulsatile tinnitus and headaches of 7 months duration. Bilateral optic disc swelling was found at fundus examination. Brain MRI excluded any brain tumor and lumbar puncture showed cerebrospinal fluid (CSF) opening pressure of 26 cm of water with normal CSF contents.

**Diagnoses:**

Idiopathic ICH was suspected.

**Interventions:**

The patient was managed with oral acetazolamide. Headaches initially improved but the dosage could not be decreased under 750 mg a day without recurrence of the symptoms. Extensive review of systems showed that the patient had active UC. He was given oral mesalazine, 2000 mg a day.

**Outcomes:**

The symptoms of UC and ICH quickly resolved. Acetazolamide was progressively tapered over the course of the 9 subsequent months and the patient did not show any worsening of his symptoms or papilledema.

**Lessons:**

UC should be added to the list of disorders associated with ICH. In case of atypical ICH with drug dependency, investigations should seek for UC. Treating efficiently UC with mesalazine may improve ICH, suggesting an underlying inflammatory process.

## 1 Introduction

Bilateral optic disc edema is the most common clinical feature of intracranial hypertension (ICH). In overweight women with normal cerebrospinal fluid (CSF) contents, elevated intracranial pressure is deemed idiopathic. The diagnosis is made after excluding other causes of ICH. Brain imaging shows signs of ICH with no mechanical obstacle impairing CSF absorption. The risk of severe and permanent visual loss persists as long as the optic disc is swollen. In some cases, ICH can be associated with inflammatory bowel disease (IBD),^[1,2]^ or triggered by medications used in the management of such disorders, for example, sulfasalazine, mesalazine, or withdrawal of corticosteroid therapy.^[3–8]^ Hereinafter, we report the unusual case of a male patient with ulcerative colitis (UC) in whom ICH resolved with mesalazine therapy.

## 2 Case report

**Figure 1:**
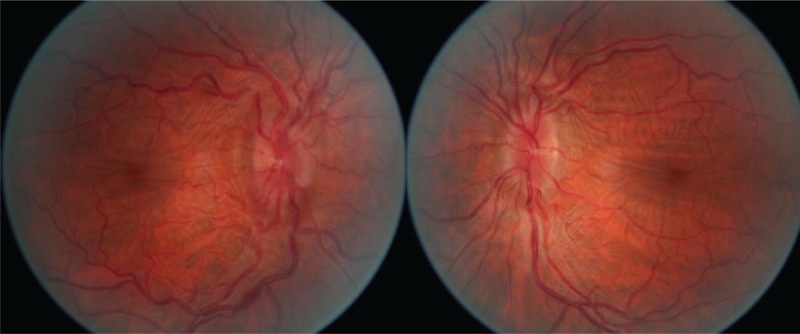
Fundus photography. Bilateral papilledema grade 2 and macular folds.

**Figure 2:**
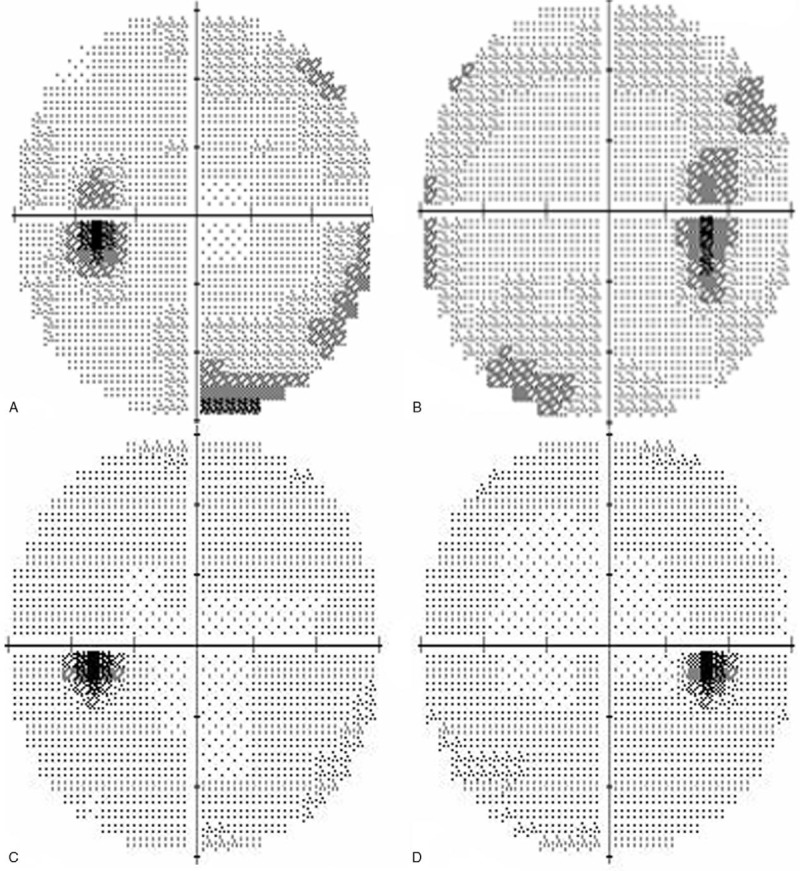
Automatized visual field perimeter 30 central degrees. a and b: Right and left eye in May 2016: enlarged blind spots and peripheral deficits. c and d: Right and left eye in March 2018: normal visual fields.

A 48-year-old Caucasian man presented to the Neuro-Ophthalmology Department of the Rothschild Ophthalmic Foundation in June 2016 for decreased vision, transient visual obscuration, pulsatile tinnitus, and headaches. His symptoms had been progressively worsening for the previous 7 months. The patient was a pharmacist and was not overweight. He had a history of UC in 2002 that was diagnosed during the workup of chronic diarrhea. The patient did not have any medication for UC. He did not smoke, nor did he use recreational drugs. On examination, best-corrected visual acuity was 20/40 OD and 20/20 OS. Eyelid examination was unremarkable. The pupils were equal and reactive. There was no relative afferent pupillary defect. Ocular movements were full and he was orthophoric in all gaze directions. Dilated fundus examination showed retinal folds between the macula and the optic disk and bilateral grade-2 optic disc swelling (Fig. 1). Visual field testing revealed enlarged blind spots and diffusely decreased visual field sensitivity in both eyes (Fig. 2). Ocular ultrasonography revealed enlarged optic nerve sheaths. Brain MRI ruled out brain tumor and venous thrombosis. There were signs of ICH including empty sella and bilateral lateral venous sinus stenosis. Lumbar puncture showed CSF opening pressure of 26 cm of water with normal CSF contents. The headaches persisted after lumbar puncture. Ancillary testing did not reveal vitamin A deficiency or anemia. Idiopathic ICH was suspected. The patient was treated with a daily dose of 1000 mg of acetazolamide that was gradually tapered. Headaches initially improved but the treatment could not be decreased under 750 mg a day without recurrence of the symptoms.

Extensive review of systems showed that patient still had chronic diarrhea. Because of the history of UC, a colonoscopy was performed in June 2017 and showed active proctitis. Fecal calprotectin level, which has been shown to be correlated to acute inflammation in intestinal inflammatory diseases, was increased to 1755 μg/mg (normal range: 10–50 μg/mg). In September 2017, he was given oral mesalazine, 2000 mg a day. UC symptoms quickly resolved, and fecal calprotectin decreased to 7 μg/mg. Symptoms of ICH and papilledema resolved in September 2017. Acetazolamide was progressively tapered over the course of the 9 subsequent months and the patient did not show any worsening of symptoms or papilledema. In September 2018, 1 month after acetazolamide withdrawal, visual acuity was 20/20 on both eyes and visual fields were full. There remained macular folds that caused metamorphopsia in the right eye. Brain MRI showed resolution of empty sella but there was persistence of bilateral lateral venous sinus stenosis.

## 3 Discussion

This case illustrates an uncommon association of ICH and UC that resolved with mesalazine.

Some studies have reported acute onset of ICH in association with acute cerebral venous thrombosis in IBD.^[9]^ To our knowledge, few cases of ICH associated with UC have been reported. All of them occurred in patients with active UC, either after discontinuation of steroids or after mesalazine introduction, suggesting that an underlying inflammatory process might be involved in the pathophysiology of ICH.^[3–5,7]^ Rosa et al (2003) reported the case of a 23-year-old woman with UC and ICH related to mesalazine use. ICH symptoms resolved after discontinuation of the drug and recurred when treatment was restored. In this case, mesalazine withdrawal was associated with the use of dexamethasone, which could have had beneficial anti-inflammatory effect. Rottembourg et al (2001) reported the case of an 11-year-old girl with Crohn disease who had ICH that resolved when mesalazine was stopped. Martin Rodriguez et al, 2007, reported the case of a 32-year-old woman with UC and mesalazine-related ICH.^[10]^ Yet, the patient also had severe anemia, which could have triggered ICH. The underlying mechanism of ICH in those latter cases remains unclear. We hypothesize that systemic inflammation associated with active UC plays a role in the pathogenesis of ICH, as suggested, in our case, by the improvement of symptoms and decreased fecal calprotectin under mesalazine. Thus, treating efficiently UC would improve ICH, in line with the cases of relapsing ICH upon steroids withdrawal. This is also supported by the case reported by Chebli et al, 2004, of severe UC, despite azathioprine, sulphazaline, and 5-ASA enema, which improved with the use of cyclosporine. Moreover, it has been stated that increased resistance of sinus venous outflow could be a crucial predisposing factor in the pathogenesis of ICH syndrome.^[11]^ Yet, a trigger factor might be required.^[11]^ In the current case, the cerebral lateral venous sinus stenosis associated with the inflammatory state related to UC might have triggered ICH. Mesalazine therapeutic mechanisms remain unknown, but this drug is believed to work through modulation of the chemical mediators of inflammatory response, particularly prostaglandins and leukotrienes. In the current case, mesalazine might have improved both UC and ICH owing to its action on the underlying inflammatory condition.

Further prospective studies are needed to improve our understanding of the pathogenic factors involved in patients with UC and ICH.

## 4 Conclusion

This case illustrates the critical need for extensive workup in patients with symptoms of ICH and normal imaging; specifically in men and in the absence of recent rapid weight gain. Although the mechanism of ICH in IBD remains unclear, there are sources of evidence that these patients are at risk of developing ICH and should be monitored accordingly. On the other hand, IBD should be considered in patients with ICH. We recommend a systematic ophthalmological examination for patients with UC presenting with any symptoms of ICH since appropriate treatment of UC could improve both conditions.

## Author contributions

**Conceptualization:** Raoul Kanav Khanna, Vanessa Polin, Thomas Sené, Catherine Vignal-Clermont.

**Data curation:** Raoul Kanav Khanna, Vanessa Polin.

**Supervision:** Raoul Kanav Khanna, Rabih Hage, Alexandre Hage, Catherine Vignal-Clermont.

**Validation:** Raoul Kanav Khanna, Rabih Hage, Alexandre Hage, Thomas Sené, Catherine Vignal-Clermont.

**Visualization:** Raoul Kanav Khanna, Rabih Hage, Alexandre Hage, Thomas Sené, Catherine Vignal-Clermont.

**Writing – original draft:** Raoul Kanav Khanna.

**Writing – review & editing:** Raoul Kanav Khanna, Rabih Hage, Alexandre Hage, Thomas Sené, Catherine Vignal-Clermont.

Raoul Kanav Khanna orcid: 0000-0001-6032-1488.
